# A Novel Departure in Advertising

**Published:** 1887-10

**Authors:** 


### *A NOVEL DEPARTURE IN ADVERTISING.*

Believing that the advertising of medicinal preparations often fails of its purpose, viz., to clearly and intelligently present to physicians their special advantages, pharmacal or therapeutic, on account of the fragmentary and imperfect manner in which the facts are usually conveyed in such advertisements, Parke, Davis & Co. propose to inaugurate rather a novel departure in advertising. It is their intention to publish in the advertising pages they occupy in medical journals a series of what they term plain talks to physicians, in each issue, taking up a certain class of preparations and pointing out the reasons why they deserve to be prescribed, until all their preparations shall have thus been presented. The excellence of the products of this house are well known, and it is to be presumed that their long experience in the manufacture of medicines will enable them to say in these informal talks something of real interest and benefit to their medical friends.

